# Knowledge and attitudes of gout patients and their perspectives about diagnosis and management: A cross‐sectional study in Saudi Arabia

**DOI:** 10.1002/iid3.1010

**Published:** 2023-09-29

**Authors:** Abdulrahman A. M. Khormi, Abdulaziz A. Basalem, Alhaytham M. Z. Al Muaddi, Abdulaziz M. Alaskar, Rakan A. S. Algahtani, Abdulsalam S. Alharbi, Tariq D. M. Alanazi, Nawaf A. Alqahtani, Abdulrahman A. S. Altamimi

**Affiliations:** ^1^ Department of Medicine Prince Sattam University Medical College Al‐Kharj Saudi Arabia; ^2^ Department of Medicine Prince Sattam Bin Abdulaziz University Al‐Kharj Saudi Arabia

**Keywords:** attitude, gout, patient, perspectives, Saudi Arabia

## Abstract

**Background:**

Gout is a chronic noncommunicable disease that might lead to multiple systemic complications if it is left untreated. The knowledge, attitudes, and perceptives among patients toward the diagnosis and management of gout are important indicators in determining the prognosis and predicting sequelae of the disease. This cross‐sectional survey aimed to assess the knowledge, attitudes, and perspectives of patients diagnosed with gout toward the disease diagnosis and treatment.

**Methodology:**

An observational cross‐sectional study was conducted at university clinics and local health facilities in central Riyadh, Saudi Arabia, for the duration between April and August 2022. Pearson *χ* ^2^ test was used to determine the difference in the proportion of patients who adapt different attitudes and perspectives in terms of their demographic variables. Statistical significance was defined as a *p* value less than .05.

**Results:**

Two‐hundred thirteen patients were involved in this study. The majority of the patients (84.0%) were diagnosed for more than 1 year. The majority of the patients (76.5%) were aged 25–60 years when they were diagnosed with gout. The most common complaint at the time of the diagnosis was joint pain (73.7%). The most commonly reported gout medication treatment being used was allopurinol accounting for 23.0%. The majority of the patients (83.6%) were satisfied regarding the effects of gout management on their job performance, work life, and careers. The vast majority (97.5%) reported that they are satisfied with the health service provided.

**Conclusion:**

The patients diagnosed with gout in Saudi Arabia exhibited a satisfactory level of information, attitude, and perspectives regarding their condition. The participants expressed a significant degree of satisfaction with the impact of gout management on their occupational performance, work‐life balance, and professional plans. Additional research is necessary to ascertain the risk factors associated with gout and provide suitable preventative interventions.

## 1 INTRODUCTION

Gout has long been recognized as being among the most common chronic inflammatory joint diseases. It is also well established that men are far more likely than women to develop gout,^1^ which is characterized by elevated serum uric acid levels (hyperuricemia), with values as high as 6.8 mg/dL. Urate crystals are formed as a result of rising blood uric acid levels, which also increases the risk of kidney stone development. Gout can occasionally be accompanied by tophi, which can eventually cause gouty arthritis.^2^ Along with a few other symptoms, acute gout can cause intense pain, swelling, and discomfort around the joints. Intercritical gout refers to the asymptomatic intervals between gout attacks. The word “podagra,” which describes a condition wherein the first metatarsophalangeal joints are impacted by urate crystals—causing severe pain—is commonly used to describe acute gout. Additionally, clearly apparent indicators of flare‐ups accompany severe gout symptoms.^2^ This includes severe inflammation that causes discomfort and pain that lasts for around 5–10 days. Asymptomatic hyperuricemia, however, persists for years in association with intermittent flare‐ups. On the other hand, the crystals can show signs of proliferation coupled with inflammation and excruciating pain, which finally enters a stage when tophi and chronic gout occur. Tophi can be seen in a variety of body sites, including articular spaces, cutaneous tissues, and bones.^3^, ^4^ People with gout may have discomfort that interferes with daily tasks, with mobility impairments that may be temporary or permanent. Quality of life is thereby significantly impacted. Stroke, diabetes, myocardial infarction, and hypertension, among other conditions, are highly associated with gout.^5^ The incidence and prevalence of gout have been rising at increasingly rapid rates over the past several years.^6^ Previous research has shown that patients often receive minimal instruction on modifying their lifestyles and adhering to their medication.^7^, ^8^

To enhance the management of gout, multiple research has been conducted to get insights into patients' knowledge, perspectives, beliefs, and obstacles pertaining to gout therapy.^9^, ^10^, ^11^, ^12^, ^13^, ^14^, ^15^, ^16^ Patient barriers encompass several factors. First, there is a lack of adequate knowledge regarding gout, which results in misconceptions about its severity and chronic nature, confusion regarding treatment options, and misunderstandings about dietary considerations. Second, patients may experience negative interactions with healthcare providers, which can hinder effective communication and impede the delivery of appropriate care. Third, patients may have negative experiences with urate‐lowering therapy, such as being unaware of the possibility of gout flares when initiating this treatment. Lastly, some patients may exhibit reluctance toward long‐term medication adherence.^12^

A previous study in Saudi Arabia by Al‐Arfaj estimated that the prevalence rate of gout is around 8.4%.^17^ There are limited studies in Saudi Arabia that examined attitudes and perspectives of gout patients.^18^, ^19^ A previous study by Alenazi et al. examined family medicine residents' knowledge, attitude, and practices toward gout in Saudi Arabia and identified a moderate level of knowledge and attitude toward the disease.^18^ Another study by Alraqibah et al. in Saudi Arabia reported suboptimal practices of healthcare providers in the management of gout patients.^19^ This study examined the knowledge, attitudes, and perceptives of gout patients toward their disease in the Kingdom of Saudi Arabia. A greater understanding of these concerns will facilitate efforts to improve gout‐related attitudes and quality of life among patients. Examining the knowledge, attitudes, and perspectives of individuals diagnosed with gout, particularly in relation to the diagnosis and management of the condition within the framework of inflammation or immunity, holds significant importance in ensuring the provision of optimal care and management for patients. This research endeavor also contributes to the early identification and intervention of the disease, adherence to treatment protocols, and enhancement of overall quality of life.

## 2 METHODOLOGY

### 2.1 Study design

This was a descriptive cross‐sectional study that was conducted on gout patients referred to university clinics and local health facilities in central Riyadh, Saudi Arabia, for the duration between April and August 2022.

### 2.2 Study population and recruitment

Patients who are currently living in Saudi Arabia, diagnosed by their physician with gout with blood uric acid levels >6 mg/dL for females and >7 mg/dL for males, and aged 18 years and above were included in this study. Any patients who did not meet the inclusion criteria, diagnosed with chronic illnesses other than gout, or did not provide consent for participation were excluded from the study.

### 2.3 Sampling procedure

The sample for this research was selected using a method referred to as convenience sampling. The present study involved individuals who voluntarily participated and met the specified inclusion criteria, afterward being determined as eligible. At the beginning of the questionnaire, patients were provided with an informed consent form and were afforded the opportunity to either proceed or discontinue their participation in the study. To facilitate the patients' understanding of the importance of their involvement, the study's aims were thoroughly elucidated. The study's invitation letter outlined the required inclusion criteria.

### 2.4 Study questionnaire

The questionnaire tool for this study was constructed based on a previous literature review.^5^, ^18^, ^20^, ^21^, ^22^ The first section was where patients indicated their consent and willingness to take part in the study. The second section captured the following demographic details: gender, age, nationality, chronic disease history, education level, and employment status. The third section collected clinical data and disease profiles from gout patients. The body mass index (BMI), prior gout episodes, disease history, and medication use were all taken into consideration. The fourth section evaluated the patients' general attitudes and practices related to gout. In addition, patients' satisfaction with health services, ability to carry out the demands and chores of daily life, sleep quality, and support from family and friends were investigated.

### 2.5 Questionnaire reliability and piloting

A preliminary investigation was undertaken by the researcher on a sample of 15 individuals who satisfied the predetermined criteria to validate their comprehension of the survey instrument and ascertain its alignment with the intended construct being assessed. The participants verified the content and face validity of the questionnaire. The questionnaire's Cronbach's *α* value of .72 indicated its reliability. Cronbach's *α* was calculated to assess the reliability of the data regarding the gout patients' perspectives on their disease and treatments in Saudi Arabia.

### 2.6 Statistical analysis

The Statistical Package for Social Science software for Windows, version 22 (IBM Corp.) was used to analyze the data for this study. Frequencies and percentages were used to present categorical variables in this study. Pearson *χ* ^2^ test or Fisher's exact test (if less than 10 observations) were used to determine the difference in the proportion of patients with “uncontrolled gout,” which we defined as “those patients reporting two or more chronic symptoms that required medical care,” and controlled gout—“those patients reporting one or no chronic symptoms” in terms of their attitude and perspectives. Statistical significance was defined as *p* value less than .05. Overall, there was a low rate of missing data (<3.8%). We excluded missing values from the analysis. Initial analyses were performed using descriptive statistics.

## 3 RESULTS

### 3.1 Demographic and clinical characteristics

Two‐hundred thirteen questionnaires were completed by adult patients currently diagnosed with gout and receiving medical treatment in the Kingdom of Saudi Arabia. The demographic characteristics of the participants are available in Table 1. More than half of the patients were aged above 51 years (77.0%) and males (64.3%). The vast majority of the patients (93.0%) were Saudis. The BMI for the vast majority of the patients (94.8%) was between 25 and 30 kg/cm^2^. The most common comorbidity among the patients was dyslipidemia accounting for 22.1%. Almost half of the patients (49.3%) reported that they hold bachelor's degrees and are full‐time workers (48.4%). There was a statistically significant difference between uncontrolled and controlled patients with gout in terms of their age and chronic disease history (*p* < .05). Patients with uncontrolled disease tend to be older and more obese.

**Table 1:** Demographic and clinical characteristics.

Variable	Category	Overall	How many recurring attacks of gout annually in your case “after” you started treatment?		*p* Value
			Uncontrolled > 2 attacks	Controlled < 2 attacks	
Age	<20	5 (2.3%)	1 (0.5%)	4 (1.9%)	<.002
	20–40	40 (18.8%)	18 (8.5%)	22 (10.3%)	
	41–50	4 (1.9%)	3 (1.4%)	1 (0.5%)	
	≥51	164 (77.0%)	109 (51.2%)	55 (25.8%)	
Sex	Male	137 (64.3%)	57 (26.8%)	80 (37.6%)	.231
	Female	76 (35.7%)	25 (11.7%)	51 (23.9%)	
Nationality	Saudi	198 (93.0%)	78 (36.6%)	120 (56.3%)	.432
	Non‐Saudi	15 (7.0%)	4 (1.9%)	11 (5.1%)	
BMI	BMI < 25	8 (3.8%)	5 (2.3%)	3 (1.4%)	.384
	BMI = 25–30	202 (94.8%)	123 (57.7%)	79 (37.1%)	
	BMI > 30	3 (1.4%)	3 (1.4%)	0 (0.0%)	
Chronic diseases (patients were prompted to select all that applied)	DM	22 (10.3%)	12 (5.6%)	10 (4.7%)	<.041
	HTN	33(15.5%)	12 (5.6%)	21 (9.9%)	
	Obesity	43 (20.2%)	32 (15.0%)	11 (5.1%)	
	Dyslipidemia	47 (22.1%)	23 (10.8%)	14 (6.6)	
	OA	6 (2.8%)	1 (0.5%)	5 (2.3%)	
	No chronic illnesses	29 (13.6%)	12 (5.6%)	17 (8.0%)	
	Other	43 (20.2%)	12 (5.6%)	31 (14.55)	
Education level	Bachelor	105 (49.3%)	67 (31.5%)	38 (17.8%)	.341
	Diploma	20 (9.4%)	11 (5.2%)	9 (4.2%)	
	Master	15 (7.0%)	9 (4.2%)	6 (2.8%)	
	PhD	5 (2.3%)	3 (1.4%)	2 (0.9%)	
	Secondary	41 (19.2%)	24 (11.3%)	17 (8.0%)	
	Noneducation	5 (2.3%)	2 (0.9%)	3 (1.4%)	
	Professor	2 (0.9%)	1 (0.5%)	1 (0.5%)	
	Others	10 (4.7%)	7 (3.3%)	3 (1.4%)	
Job status	Full‐time	103 (48.4%)	55 (25.8%)	48 (22.5)	.052
	Nonemployed	40 (18.8%)	25 (11.7%)	15 (7.0)	
	Part‐time	11 (5.2%)	7 (3.3%)	4 (1.9%)	
	Retired	25 (11.7%)	17 (8.0%)	8 (3.8)	
	Student	21 (9.9%)	17 (8.0%)	4 (1.9%)	

Abbreviations: BMI, body mass index; DM, diabetes mellitus; HTN, hypertension; OA, osteoarthritis.

### 3.2 Time of diagnosis and medical personnel who diagnosed the patients

Table 2 below presents a time of diagnosis and the medical personnel who diagnosed the patients. The majority of the patients (84.0%) were diagnosed for more than 1 year. The majority of the patients (76.5%) were aged 25–60 years when they were diagnosed with gout. Almost one‐third of the patients (32.4%) were diagnosed with gout 6–12 months after the onset of their symptoms. Almost one‐third of the patients (33.3%) were diagnosed by their family physician. There was a statistically significant difference between uncontrolled and controlled patients with gout in terms of their age at diagnosis, duration between symptoms and diagnosis, and medical personnel who diagnosed them (*p* < .05). A highest proportion of the patients with uncontrolled disease were diagnosed between the age 25 and 60 years, diagnosed with gout 6–12 months after the onset of their symptoms, and diagnosed by their family physician.

**Table 2:** Time of diagnosis and the medical personnel who diagnosed the patients.

Questionnaire	Response options	How many recurring attacks of gout annually in your case “after” you started treatment?			*p* Value
		Overall	Uncontrolled > 2 attacks	Controlled < 2 attacks	
How many years ago were you diagnosed with gout?	Less than 1	34 (16.0%)	20 (9.4%)	14 (6.6%)	.632
	1 or more	179 (84.0%)	111 (52.1)	68 (31.9%)	
How old were you when someone diagnosed you with gout?	15–25	40 (18.8%)	24 (11.3%)	16 (7.5%)	.043
	25–60	163 (76.5%)	100 (46.9%)	63 (29.6%)	
	>60	10 (4.7%)	7 (3.3%)	3 (1.4%)	
How much time elapsed between the onset of symptoms and your diagnosis of gout (months)?	1–3 months	68 (31.9%)	41 (19.2%)	27 (12.7%)	.043
	3–6 months	69 (32.4%)	45 (21.1%)	24 (11.3%)	
	6–12 months	47 (22.1%)	32 (15.0%)	15 (7.0%)	
	>12 months	29 (13.6%)	13 (6.1%)	16 (7.5%)	
	Rheumatologist	46 (21.6%)	36 (16.9%)	10 (4.7%)	.027
	Internist	29 (13.6%)	17 (8.0%)	12 (5.6%)	
Who diagnosed you with gout?	Family physician	71 (33.3%)	39 (18.3%)	32 (15.0%)	.027
	Orthopedic surgeon	41 (19.2%)	23 (10.8%)	18 (8.5%)	
	Unknown	23 (10.8%)	14 (6.6%)	9 (4.2%)	
	Other	3 (1.4%)	2 (0.9%)	1 (0.5%)	

### 3.3 Clinical picture, investigations, and management

Table 3 below presents the clinical picture, investigations, and management profile for the patients. Almost half of the patients (51.2%) reported that they are currently suffering from gout attacks. The most common complaint at the time of the diagnosis was joint pain (73.7%). More than half of the patients (57.3%) reported that they had two recurring attacks of gout annually before they started treatment. The most commonly reported gout medication treatment being used was allopurinol accounting for 23.0%. The most commonly undergone tests were thyroid function test (TFT) and C‐reactive protein(CRP) accounting for 14.6% each. More than half of the patients (54.0%) reported that they did not visit a nutritionist to treat their disease. There was a statistically significant difference between uncontrolled and controlled patients with gout in terms of most common complaint at the time of diagnosis, frequency of annual attacks of gout before starting treatment, gout medications used, tests performed, and referring to nutritionist to treat their disease (*p* < .05). Patients with uncontrolled disease were more likely to have joints pain, higher number of attacks, higher proportion of medications use, higher proportion of tests being performed, and more likely to visit nutritionist to treat their disease.

**Table 3:** Clinical picture, investigations, and management.

Questionnaire	Response options	How many recurring attacks of gout annually in your case “after” you started treatment?			*p* Value
		Overall	Uncontrolled > 2 attacks	Controlled < 2 attacks	
Are you currently suffering from a gout attack? (*n* = 187)	No	109 (51.2%)	68 (31.9%)	41 (19.2%)	.532
	Yes	78 (36.6%)	49 (23.0%)	29 (13.6%)	
At the time of diagnosis, can you choose more than one complaint?	Joints pain	157 (73.7%)	95 (44.6%)	62 (29.1%)	<.022
	Tophi	11 (5.2%)	3 (1.4%)	8 (3.8%)	
	Kidney disease	7 (3.3%)	6 (2.8%)	1 (0.5%)	
	Asymptomatic high uric acid	18 (8.5%)	12 (5.6%)	6 (2.8%)	
	Other	20 (9.4%)	8 (3.8%)	12 (4.7%)	
How many recurring attacks of gout annually in your case “before” you started treatment? (*n* = 209)	1–2	29 (13.6%)	18 (8.5%)	11 (5.2%)	.032
	2	122 (57.3%)	69 (32.4%)	53 (24.9%)	
	>2	58 (27.2%)	40 (18.8%)	18 (8.5%)	
Please choose the current gout medications you are using (you can choose more than one option)	Colchicine	43 (20.2%)	24 (11.3%)	19 (8.9%)	<.012
	Allopurinol	49 (23.0%)	28 (13.1%)	21 (9.9%)	
	Probenecid	29 (13.6%)	15 (7.0%)	14 (6.6%)	
	Febuxostat	25 (11.7%)	14 (6.6%)	11 (5.2%)	
	NSAIDs	18 (8.5%)	12 (5.6%)	6 (2.8%)	
	Steroids	9 (4.2%)	6 (2.6%)	3 (1.4%)	
	None	23 (10.8%)	17 (8.0%)	6 (2.8%)	
	Other	17 (8.0%)	10 (4.7%)	7 (3.3%)	
Which of the following tests have you undergone (you can use more than one option)	RFT	23 (10.8%)	11 (5.2%)	12 (5.6%)	<.042
	LFT	19 (8.9%)	10 (4.7%)	9 (4.2%)	
	CBC	14 (6.6%)	13 (6.1%)	1 (0.5%)	
	ESR	4 (1.9%)	3 (1.4%)	1 (0.5%)	
	CRP	31 (14.6%)	12 (5.6%)	19 (8.9%)	
	TFT	31 (14.6%)	20 (9.4%)	11 (5.2%)	
	X‐ray	9 (4.2%)	5 (2.3%)	4 (1.9%)	
	MSK US	7 (3.3%)	4 (1.9%)	3 (1.4%)	
	CT	12 (5.6%)	9 (4.2%)	3 (1.4%)	
	Joints aspiration	9 (4.2%)	6 (2.6%)	3 (1.4%)	
	Uric acid level	8 (3.8%)	8 (3.8%)	0 (0.0%)	
	Unknown	17 (8.0%)	10 (4.7%)	7 (3.3%)	
	None	13 (6.1%)	9 (4.2%)	4 (1.9%)	
	Other	16 (7.5%)	6 (2.8%)	10 (4.7%)	
Have you visited a nutritionist to treat gout?	No	115 (54.0%)	65 (30.5%)	50 (23.5%)	<.037
	Yes	98 (46.0%)	66 (31.0%)	32 (15.0%)	

Abbreviations: CBC, complete blood count; CRP, C‐reactive protein; CT, computed tomography; ESR, erythrocyte sedimentation rate; LFT, liver function test; MSK US, musculoskeletal ultrasound; NSAIDs, nonsteroidal anti‐inflammatory drugs; RFT, renal function test; TFT, thyroid function test.

### 3.4 Medical education and lifestyle programs

Table 4 presents patients' medical education and lifestyle. The vast majority of the patients (90.1%) confirmed that they discussed with their doctor about not eating excessively foods that contain high levels of uric acid and the ideal level of uric acid in the blood that should be achieved after treatment for gout (85.0%). The majority of the patients (74.2%) confirmed that they discussed with their doctor how to treat acute gout attacks and medications that can lower the level of uric acid in the blood (68.1%). Around one‐quarter the patients (25.4%) reported that they use painkillers to treat their attacks. More than half of the patients (69.5%) confirmed that they discussed with their doctor how long they should continue taking the treatment and about adopting a healthy lifestyle (68.1%). Less than half of the patients (45.1%) confirmed that they discussed with their doctor about stopping smoking and about losing weight and following a healthy lifestyle (42.7%). Almost one‐third of the patients (35.2%) reported that they should eat less meat to take control of their disease. There was a statistically significant difference between uncontrolled and controlled patients with gout in terms of discussing how to treat acute gout attacks, medication use, and adopting a healthy lifestyle (*p* < .05).

**Table 4:** Medical education and lifestyle programs.

Questionnaire prompt	Response options	Overall	How many recurring attacks of gout annually in your case “after” you started treatment?		*p* Value
			Uncontrolled > 2 attacks	Controlled < 2 attacks	
Have you discussed with your doctor about not eating excessively foods that contain high levels of uric acid, such as: meat, legumes?	No	21 (9.9%)	9 (4.2%)	12 (5.6%)	.532
	Yes	192 (90.1%)	118 (55.4%)	74 (34.7%)	
Have you discussed with your doctor the ideal level of uric acid in the blood that should be achieved after treatment for gout for gout?	No	32 (15.0%)	21 (9.9%)	11 (5.2%)	.213
	Yes	181 (85.0%)	110 (51.6%)	71 (33.3%)	
Have you discussed with your doctor how to treat acute gout attacks?	No	24 (11.3%)	11 (5.2%)	13 (6.1%)	.043
	Yes	158 (74.2%)	100 (46.9%)	58 (27.2%)	
Have you discussed with your doctor medications that can lower the level of uric acid in the blood?	No	68 (31.9%)	33 (15.5%)	35 (16.4%)	.049
	Yes	145 (68.1%)	98 (46.0%)	47 (22.1%)	
What do you do to treat acute gout attacks (you can choose more than one option)?	More fluids	53 (24.9%)	31 (14.6%)	22 (10.3%)	.731
	Painkillers	54 (25.4%)	35 (16.4%)	19 (8.9%)	
	Colchicine	47 (22.1%)	32 (15.0%)	15 (7.0%)	
	Steroid	29 (13.6%)	13 (6.1%)	16 (7.5%)	
	Others	30 (14.1%)	20 (9.4%)	10 (4.7%)	
Have you discussed with your doctor how long you should continue taking the treatment?	No	30 (14.1%)	15 (7.0%)	15 (7.0%)	.354
	Yes	148 (69.5%)	94 (44.1%)	54 (25.4%)	
Have you discussed with your doctor about adopting a healthy lifestyle such as eating moderate amounts of red meat, legumes and shrimp, to reduce the level of uric acid in the blood in addition to medication?	No	68 (31.9%)	33 (15.5%)	35 (16.4%)	.049
	Yes	145 (68.1%)	98 (46.0%)	47 (22.1%)	
Have you discussed with your doctor about stopping smoking?	No	117 (54.9%)	63 (29.6%)	54 (25.4%)	.563
	Yes	96 (45.1%)	35 (16.4%)	61 (28.6)	
Have you discussed with your doctor about losing weight and following a healthy lifestyle?	No	122 (57.3%)	61 (28.6)	61 (28.6)	.754
	Yes	91 (42.7%)	41 (19.2%)	50 (23.5%)	
Which of the following lifestyle measures should you take to control gout? (You can choose more than one option)	less red meat consumption	75 (35.2%)	32 (15.0%)	43 (20.2%)	.375
	Less sea food	39 (18.3%)	16 (7.5%)	23 (10.8%)	
	Less legumes and beans	31 (14.6%)	12 (5.6%)	19 (8.9%)	
	More fluid	34 (16.0%)	22 (10.3%)	12 (5.6%)	
	Stop smoking	15 (7.0%)	12 (5.6%)	3 (1.4%)	
	Sports	12 (5.6%)	8 (3.8%)	4 (1.9%)	
	Weight loss	7 (3.3%)	5 (2.3%)	2 (0.9%)	

### 3.5 Satisfaction with healthcare services

Table 5 below presents patients' satisfaction with healthcare services. Patients with uncontrolled diseases were more likely to be satisfied compared to controlled patients in terms of health service provided, ability to carry out the demands and chores of daily life since diagnosis, and support from family and friends (*p* < .05).

**Table 5:** Satisfaction with healthcare services.

Questionnaire prompt	Response options	How many recurring attacks of gout annually in your case “after” you started treatment?		*p* Value
		Uncontrolled > 2 attacks	Controlled < 2 attacks	
How satisfied are you the health service provided to you?	Very dissatisfied	0 (0.0%)	1 (0.5%)	.043
	Dissatisfied	6 (2.8)	6 (2.8%)	
	Neutral	7 (3.29%)	15 (7.0%)	
	Satisfied	45 (21.1%)	24 (11.3%)	
	Very satisfied	73 (34.2%)	36 (16.9)	
How satisfied are you with ability to carry out the demands and chores of daily life since diagnosis?	Dissatisfied	11 (5.2)	11 (5.2%)	.051
	Neutral	22 (10.3%)	17 (8.0%)	
	Satisfied	57 (26.8%)	32 (15.0%)	
	Very satisfied	41 (19.2%)	22 (10.3%)	
How satisfied are you with sleep quality?	Very dissatisfied	1 (0.5%)	0 (0.0%)	.154
	Dissatisfied	12 (5.6%)	15 (7.0%)	
	Neutral	32 (15.0%)	23 (10.8%)	
How satisfied are you with support from family and friends?	Very dissatisfied	1 (0.5%)	0 (0.0%)	.032
	Dissatisfied	9 (4.2%)	7 (3.3%)	
	Neutral	12 (5.6%)	18 (8.5%)	
	Satisfied	29 (13.6%)	22 (10.3%)	
	Very satisfied	80 (37.6%)	35 (16.4%)	

Table 6 summarizes the patient quality‐of‐life assessment. The majority of the patients (83.6%) were satisfied regarding the effects of gout management on their job performance, work life, and careers. Around half of the patients (53.1%) reported that they are satisfied with the effects of gout on demands and chores of daily life since their diagnosis. Around 61.1% of the patients reported that they were satisfied with their sleep quality. The majority of the patients (77.9%) reported that they are satisfied with support from family and friends and the vast majority (97.5%) reported that they are satisfied with the health service provided.

**Table 6:** Patient quality of life assessment about gout.

Questionnaire prompt	Very dissatisfied	Dissatisfied	Neutral	Satisfied	Very satisfied
How satisfied are you with the effects of gout on your job performance, work life, and career?	1 (0.5%)	12 (5.6%)	22 (10.3%)	69 (32.4%)	109 (51.2%)
How satisfied are you with the effects of gout on demands and chores of daily life since your diagnosis?		34 (16%)	66 (31%)	50 (23.5%)	63 (29.6%)
How satisfied are you with your sleep quality?	1 (0.5%)	27 (12.7%)	55 (25.8)%	70 (32.9%)	60 (28.2%)
How satisfied are you with your support from family and friends	1 (0.5%)	16 (7.5%)	30 (14.1%)	51 (23.9%)	115 (54%)
How satisfied are you with health service provide for you	(0.1%)	18 (7.00%)	1507.1%)	61 (33.5%)	125 (64%)

## 4 DISCUSSION

The aim of this study was to evaluate the knowledge, attitudes, and perspectives of individuals diagnosed with gout on the diagnosis and management of the disease. Our study provides information on patient knowledge and perceptions regarding gout management. Several deficits in knowledge, attitude, and perspectives were identified in the study sample, including in the quality assessment of knowledge about gout and information about gout. However, the deficits were greater in those with uncontrolled gout. The primary outcomes of this research are outlined as follows: (1) The prevailing symptom observed during the diagnostic phase was joint pain. (2) A majority of patients disclosed experiencing two recurrent episodes of gout per year before commencing treatment. (3) Allopurinol emerged as the most frequently employed medication for gout management. (4) A significant proportion of patients affirmed engaging in discussions with their healthcare provider regarding dietary restrictions pertaining to high uric acid content, as well as the target level of uric acid in the bloodstream posttreatment for gout. (5) The findings indicate that a significant proportion of the patients expressed satisfaction with respect to the impact of gout management on their job performance, work‐life balance, and professional plans. (6) Approximately half of the patients expressed satisfaction regarding the impact of gout on their daily life responsibilities and tasks subsequent to their diagnosis. Furthermore, they reported contentment with their sleep quality. (7) A significant proportion of the patients conveyed satisfaction with the support received from their family and friends, as well as with the healthcare services provided.

The majority of the patients (76.5%) were aged 25–60 years when they were diagnosed with gout. Almost one‐third of the patients (32.4%) were diagnosed with gout 6–12 months after the onset of their symptoms. In our study, males' patients were more likely to be uncontrolled compared to females. This is similar to the findings of a previous study in Pakistan.^22^ Gout is considered to be one of the earliest and most widespread manifestations of inflammatory arthritis. The prevalence of this ailment has exhibited an upward trend in recent decades, indicating a concerning issue in the field of public health. However, it has been observed that the prevalence of the disease varies throughout different parts of the world, with a greater number of cases reported in the Pacific regions.^23^ Genetic factors contribute to the pathogenesis of gout, whereas certain ethnic groups exhibit a higher susceptibility to this condition when compared to others. The combination of these factors contributes to an increasing death rate and negatively impacts the life expectancy rate.^24^

Consistent with the findings of our study, the research conducted by Ashiq et al. in Pakistan revealed that males have a fourfold greater risk compared to females.^22^ Women experience this advantage as a result of the presence of estradiol, which acts as an antagonist to the formation of urate crystals.^25^ The age range of those affected by gout was initially documented as beginning in 30s, with a majority of patients falling within this age group. The majority of patients were observed to fall between the age range of 41–45 years, suggesting that the onset of the disease does not typically occur at a young age. Gout primarily manifests in those aged 65 and older, with obesity being identified as a significant contributing factor to its onset at younger ages.^26^

In our study, the most common complaint at the time of the diagnosis was joint pain (73.7%). More than half of the patients (57.3%) reported that they had two recurring attacks of gout annually before they started treatment. Besides, the most commonly reported gout medication treatment being used was allopurinol accounting for 23.0%. In addition, around one‐quarter the patients (25.4%) reported that they use painkillers to treat their attacks. The most commonly undergone tests were TFT and CRP accounting for 14.6% each. This observation is consistent with the results of previous research.^22^, ^27^, ^28^, ^29^ The spectrum of joints afflicted by gout often spans from three to eight, resulting in the manifestation of intense discomfort.^22^ Gout is a persistent medical condition characterized by multiple stages. Over the course of many years, the length of time and individuals affected by untreated gout may go from acute gout episodes that affect one or a few joints to more regular and recurring attacks that affect multiple joints.^22^ If left untreated, gout may develop into chronic tophaceous gout. This information is supported by reference.^27^ Regarding the therapeutic approach, a considerable number of patients were administered either a singular medication or a combination of pharmaceutical agents, such as allopurinol, febuxostat, colchicine, and nonsteroidal anti‐inflammatory drugs. The administration of allopurinol, colchicine, and oral glucocorticosteroids has undergone validation and improvement, and has been determined to be a cost‐effective treatment plan for the majority of patients.^28^, ^29^

In our study, the vast majority of the patients (90.1%) confirmed that they discussed with their doctor about not eating excessively foods that contain high levels of uric acid and the ideal level of uric acid in the blood that should be achieved after treatment for gout (85.0%). Besides, the majority of the patients (83.6%) were satisfied regarding the effects of gout management on their job performance, work life, and careers. Around half of the patients (53.1%) reported that they are satisfied with the effects of gout on demands and chores of daily life since their diagnosis. Around 61.1% of the patients reported that they were satisfied with their sleep quality. The majority of the patients (77.9%) reported that they are satisfied with support from family and friends and the vast majority (97.5%) reported that they are satisfied with the health service provided. This was different from the findings of a previous study conducted in Australia,^30^ which demonstrated gout to have a significant impact on patients' lives; the findings suggested that the patients' primary concerns were poor mobility and the pain associated with gout flare‐ups. The study report also covered the social impacts of gout and its negative effects on productivity. Another study in Pakistan found similar findings to the Australian study.^22^ The findings of the study indicated that the patients expressed consensus regarding the impact of gout on their daily activities, noting that the disease imposes an additional burden on their lives.^22^ Furthermore, the patients reported experiencing limitations in movement, resulting in a reliance on others for assistance.^22^ Additionally, it was seen that the mental well‐being of all the patients was significantly compromised. The presence of acute pain, subcutaneous tophi, and prolonged low‐grade inflammation can lead to joint deformity, limited mobility, and lifelong impairment, exerting a detrimental impact on the patient's quality of life.^22^ The management of gout poses a considerable level of complexity. To optimize the management of gout, patients should possess knowledge regarding the impact of medicine, daily routine, and dietary choices on their present medical state.^22^ The lack of patient education has been identified as a potential cause for medication nonadherence and a decline in quality of life.^31^, ^32^, ^33^ The majority of patients had a preference for consulting rheumatologists rather than primary healthcare physicians for their regular examinations and sought assistance from pharmacists to enhance disease management.^31^

In addition to addressing bone‐related problems, rheumatologists are progressively inclined to engage in discussions pertaining to aspects beyond the direct pharmacological management of joint ailments, including patient quality of life and education.^34^, ^35^ The implementation of improved pharmacy services has been recognized as a means to effectively tackle issues pertaining to medications and drug‐related concerns. The involvement of pharmacists in primary health care should be further elucidated in the management of patients with gout, particularly with regard to the current provision of patient education and the monitoring of medication adherence.^36^, ^37^, ^38^

## 5 CONCLUSION

Patients with gout in Saudi Arabia showed an adequate level of knowledge, attitude, and perspectives about their disease. The patients reported a high level of satisfaction concerning the effects of gout management on their job performance, work life, and careers. In addition, patients were satisfied in terms of the health service provided. Further studies are warranted to identify risk factors of gout and recommend appropriate preventive measures.

## AUTHOR CONTRIBUTIONS

Abdulrahman A. M. Khormi conceived and designed the study, conducted research, provided research materials, and commenced writing the initial and final draft of the article. Abdulaziz A. Basalem, Alhaytham M. Z. Al Muaddi, Abdulaziz M. Alaskar, Rakan A. S. Algahtani, Abdulsalam S. Alharbi, Tariq D. M. Alanazi, Nawaf A. Alqahtani, and Abdulrahman A. S. Altamimi collected, organized, analyzed, and interpreted data. Also, they provided logistic support. All authors have critically reviewed and approved the final draft and are responsible for the content and similarity index of the manuscript.

## CONFLICT OF INTEREST STATEMENT

The authors declare no conflict of interest.

## ETHICS STATEMENT

This research had ethical approval from Prince Sattam bin Abdulaziz University's Deanship of Scientific Research, Research Ethics Committee in Health and Science Disciplines, approval number (REC‐HSD‐99‐2021). All study participants gave their informed consent for inclusion before they participated in the study.

## Supporting information

Supporting information.

Supporting information.

## Data Availability

The data that support the findings of this study are available on request from the corresponding author. The data are not publicly available due to privacy or ethical restrictions.
