# Supplementary material for: Knowledge and attitudes of gout patients and their perspectives about diagnosis and management: A cross‐sectional study in Saudi Arabia
Source: Immun Inflamm Dis. 2023 Sep 29;11(9):e1010. doi: 10.1002/iid3.1010 (PMC10540143; doi:10.1002/iid3.1010)
Supplement: Supplementary file 1 — Supporting information. [file IID3-11-e1010-s002.docx]

**Questionnaire tool:**

| Variable | Category |
| --- | --- |
|  |  |
| Age | <20 |
|  | 20-40 |
|  | 41-50 |
|  | ≥51 |
| Sex | Male |
|  | Female |
| Nationality | Saudi |
|  | Non-Saudi |
| BMI | BMI less than 25 |
|  | BMI of 25 to 30 |
|  | BMI above 30 |
| Chronic diseases  (patients were prompted to select all that applied): | DM |
|  | HTN |
|  | Obesity |
|  | Dyslipidemia |
|  | OA |
|  | No chronic illnesses |
|  | Other |
| Education level | Bachelor |
|  | Diploma |
|  | Master |
|  | PhD |
|  | Secondary |
|  | Non-Education |
|  | Professor |
|  | Others |
| Job status | Full-time |
|  | Non-employed |
|  | Part-time |
|  | Retired |
|  | Student |

*Time of Diagnosis and the Medical Personnel Who Diagnosed the Patients*

| Questionnaire | Response options |
| --- | --- |
|  |  |
| How many years ago were you diagnosed with gout? | Less than 1 |
|  | 1 or more |
| How old were you when someone diagnosed you with gout? | 15-25 |
|  | 25-60 |
|  | >60 |
| How much time elapsed between the onset of symptoms and your diagnosis of gout (months)? | 1-3 months |
|  | 3-6 months |
|  | 6-12 months |
|  | >12 months |
|  | Rheumatologist |
|  | Internist |
| Who diagnosed you with gout? | Family physician |
|  | Orthopedic surgeon |
|  | Unknown |
|  | Other |

*Clinical Picture, Investigations, and Management*

| Questionnaire | Response options |
| --- | --- |
|  |  |
| Are you currently suffering from a gout attack? | No |
|  | Yes |
| At the time of diagnosis, can you choose more than one complaint? | Joints pain |
|  | Tophi |
|  | Kidney disease |
|  | Asymptomatic high uric acid |
|  | Other |
| How many recurring attacks of gout annually in your case “before” you started treatment? | 1-2 |
|  | 2 |
|  | > 2 |
| Please choose the current gout medications you are using (you can choose more than one option) | Colchicine |
|  | Allopurinol |
|  | Probenecid |
|  | Febuxostat |
|  | NSAIDs |
|  | Steroids |
|  | None |
|  | Other |
| Which of the following tests have you undergone (you can use more than one option) | RFT |
|  | LFT |
|  | CBC |
|  | ESR |
|  | CRP |
|  | TFT |
|  | X-ray |
|  | MSK US |
|  | CT |
|  | Joints aspiration |
|  | Uric acid level |
|  | Unknown |
|  | None |
|  | Other |
| Have you visited a nutritionist to treat gout? | No |
|  | Yes |

*Medical Education and Lifestyle Programs*

| Questionnaire prompt | Response options |
| --- | --- |
|  |  |
| Have you discussed with your doctor about not eating excessively foods that contain high levels  of uric acid, such as: meat, legumes? | No |
|  | Yes |
| Have you discussed with your doctor the ideal level of uric acid in the blood that should be achieved after treatment for gout  for gout? | No |
|  | Yes |
| Have you discussed with your doctor how to treat  acute gout attacks? | No |
|  | Yes |
| Have you discussed with your doctor medications that can lower the level of uric acid in the blood? | No |
|  | Yes |
| What do you do to treat acute gout attacks (you can choose more than one option)? | More fluids |
|  | Pain killers |
|  | Colchicine |
|  | Steroid |
|  | Others |
| Have you discussed with your doctor how long you should continue taking the treatment? | No |
|  | Yes |
| Have you discussed with your doctor about adopting a healthy lifestyle such as eating moderate amounts of red meat, legumes and shrimp, to reduce the level of uric acid in the  blood in addition to medication? | No |
|  | Yes |
| Have you discussed with your doctor about stopping smoking? | No |
|  | Yes |
| Have you discussed with your doctor about losing weight and following a healthy lifestyle? | No |
|  | Yes |
| Which of the following lifestyle measures should you take to control gout? (You can  choose more than one option) | less red meat consumption |
|  | Less sea food |
|  | Less legumes and beans |
|  | More fluid |
|  | Stop smoking |
|  | Sports |
|  | Weight loss |

***Patient Quality of Life Assessment About Gout***

| **Questionnaire prompt** | **Very dissatisfied** | **Dissatisfied** | **Neutral** | **Satisfied** | **Very satisfied** |
| --- | --- | --- | --- | --- | --- |
| How satisfied are you with the effects of gout on your job performance, work life, and career? |  |  |  |  |  |
| How satisfied are you with the effects of gout on demands and chores of daily life since your diagnosis? |  |  |  |  |  |
| How satisfied are you with your sleep quality? |  |  |  |  |  |
| How satisfied are you with your support from family and friends  How satisfied are you with health service provide for you |  |  |  |  |  |
