# Supplementary material for: Knowledge and attitudes of gout patients and their perspectives about diagnosis and management: A cross‐sectional study in Saudi Arabia
Source: Immun Inflamm Dis. 2023 Sep 29;11(9):e1010. doi: 10.1002/iid3.1010 (PMC10540143; doi:10.1002/iid3.1010)
Supplement: Supplementary file 2 — Supporting information. [file IID3-11-e1010-s001.pdf]

STROBE Statement—checklist of items that should be included in reports of observational studies

	Item No.	Recommendation	Page No.	Relevant text from manuscript
**Title and abstract**	1	(*a*) Indicate the study's design with a commonly used term in the title or the abstract	4	Knowledge of patients and attitude towards diagnosis and management is an important indicator
		(*b*) Provide in the abstract an informative and balanced summary of what was done and what was found	5	
**Introduction**				
Background/rationale	2	Explain the scientific background and rationale for the investigation being reported	6	For a very long time, gout has been recognized as the most common chronic inflammatory joint diseases
Objectives	3	State specific objectives, including any prespecified hypotheses	6-7	
**Methods**				
Study design	4	Present key elements of study design early in the paper	7	the research was descriptive cross-sectional with a sample size of 213 participants . Data was gathered between April 2022 and August 2022. The data collecting questionnaire was straightforward, understandable, and indicative of the study's goal
Setting	5	Describe the setting, locations, and relevant dates, including periods of recruitment, exposure, follow-up, and data collection	7-8	
Participants	6	(*a*) *Cohort study*—Give the eligibility criteria, and the sources and methods of selection of participants. Describe methods of follow-up<br>*Case-control study*—Give the eligibility criteria, and the sources and methods of case ascertainment and control selection. Give the rationale for the choice of cases and controls<br>*Cross-sectional study*—Give the eligibility criteria, and the sources and methods of selection of participants	7-8	
		(*b*) *Cohort study*—For matched studies, give matching criteria and number of exposed and unexposed<br>*Case-control study*—For matched studies, give matching criteria and the number of controls per case		
Variables	7	Clearly define all outcomes, exposures, predictors, potential confounders, and effect modifiers. Give diagnostic criteria, if applicable	7-8	
Data sources/ measurement	8*	For each variable of interest, give sources of data and details of methods of assessment (measurement). Describe comparability of assessment methods if there is more than one group	7-8	
Bias	9	Describe any efforts to address potential sources of bias	7-8	
Study size	10	Explain how the study size was arrived at	7	

Continued on next page

Quantitative variables	11	Explain how quantitative variables were handled in the analyses. If applicable, describe which groupings were chosen and why	7-8	Gout patients with blood uric acid levels more than 6 mg/dl who reside in Saudi Arabia met the study's inclusion criteria. Only people who were prepared to contribute were taken into consideration
Statistical methods	12	(*a*) Describe all statistical methods, including those used to control for confounding	7-8	
		(*b*) Describe any methods used to examine subgroups and interactions	7-8	
		(*c*) Explain how missing data were addressed	7-8	
		(*d*) *Cohort study*—If applicable, explain how loss to follow-up was addressed<br>*Case-control study*—If applicable, explain how matching of cases and controls was addressed<br>*Cross-sectional study*—If applicable, describe analytical methods taking account of sampling strategy	7-8	
		(*e*) Describe any sensitivity analyses	7-8	
**Results**				
Participants	13*	(a) Report numbers of individuals at each stage of study—eg numbers potentially eligible, examined for eligibility, confirmed eligible, included in the study, completing follow-up, and analysed	8-20	109(51.2%) of them with uncontrolled Gout attack, 55(25.8%) of controlled gout follow by 18(8.5%) of 20-60 yrs has uncontrolled gout attack and 22 (10.3%) has controlled gout attack, likewise the P value(< 0.002) indicate significant association between annually recurring attacks of gout after treatment
		(b) Give reasons for non-participation at each stage	8-20	
		(c) Consider use of a flow diagram		
Descriptive data	14*	(a) Give characteristics of study participants (eg demographic, clinical, social) and information on exposures and potential confounders	8-20	
		(b) Indicate number of participants with missing data for each variable of interest	8-20	
		(c) *Cohort study*—Summarise follow-up time (eg, average and total amount)		
Outcome data	15*	*Cohort study*—Report numbers of outcome events or summary measures over time		
		Case-control study—Report numbers in each exposure category, or summary measures of exposure		
		Cross-sectional study—Report numbers of outcome events or summary measures	8-20	
Main results	16	(*a*) Give unadjusted estimates and, if applicable, confounder-adjusted estimates and their precision (eg, 95% confidence interval). Make clear which confounders were adjusted for and why they were included	8-20	
		(*b*) Report category boundaries when continuous variables were categorized	8-20	
		(*c*) If relevant, consider translating estimates of relative risk into absolute risk for a meaningful time period		

Continued on next page

Other analyses	17	Report other analyses done—eg analyses of subgroups and interactions, and sensitivity analyses	8-20	
**Discussion**				
Key results	18	Summarise key results with reference to study objectives	21-22	Our study provides information on patient knowledge and treatment perception regarding gout management. Several deficits in knowledge were identified in the total population including Quality assessment of Knowledge about gout, information about gout, demographic information about the patient
Limitations	19	Discuss limitations of the study, taking into account sources of potential bias or imprecision. Discuss both direction and magnitude of any potential bias	21-22	
Interpretation	20	Give a cautious overall interpretation of results considering objectives, limitations, multiplicity of analyses, results from similar studies, and other relevant evidence	21-22	
Generalisability	21	Discuss the generalisability (external validity) of the study results	21-22	
**Other information**				
Funding	22	Give the source of funding and the role of the funders for the present study and, if applicable, for the original study on which the present article is based	24	the project number (IF-PSAU-2021/03/17631

*Give information separately for cases and controls in case-control studies and, if applicable, for exposed and unexposed groups in cohort and cross-sectional studies.

Note: An Explanation and Elaboration article discusses each checklist item and gives methodological background and published examples of transparent reporting. The STROBE checklist is best used in conjunction with this article (freely available on the Web sites of PLoS Medicine at http://www.plosmedicine.org/, Annals of Internal Medicine at http://www.annals.org/, and Epidemiology at http://www.epidem.com/). Information on the STROBE Initiative is available at www.strobe-statement.org.
